# Cytotoxicity of *Eupatorium cannabinum L.* ethanolic extract against colon cancer cells and interactions with Bisphenol A and Doxorubicin

**DOI:** 10.1186/1472-6882-14-264

**Published:** 2014-07-24

**Authors:** Edna Ribeiro-Varandas, Filipe Ressurreição, Wanda Viegas, Margarida Delgado

**Affiliations:** Centro de Botânica Aplicada à Agricultura, Instituto Superior de Agronomia, Universidade de Lisboa, Tapada da Ajuda, 1349-017 Lisboa, Portugal; Faculty of Psychology and Life Sciences, Universidade Lusófona de Humanidades e Tecnologias, Campo Grande 376, 1749-024 Lisboa, Portugal

## Abstract

**Background:**

*Eupatorium cannabinum L.* has long been utilized in traditional medicine, however no information is available regarding cellular effects of full extracts*.* Here we assessed the effects of *E. cannabinum* ethanolic extract (EcEE) on the colon cancer line HT29. Potential interactions with bisphenol A (BPA) a synthetic phenolic compound to which humans are generally exposed and a commonly used chemotherapeutic agent, doxorubicin (DOX) were also evaluated.

**Methods:**

HT29 cells were exposed to different concentrations (0.5 to 50 μg/ml) of EcEE alone or in combination with BPA or DOX. Cell viability was analyzed through resazurin assay. Gene transcription levels for *NCL, FOS, p21*, *AURKA* and *bcl-xl* were determined through qRT-PCR. Cytological analysis included evaluation of nuclear and mitotic anomalies after DAPI staining, immunodetection of histone H3 lysine 9 acetylation (H3K9ac) and assessment of DNA damage by TUNEL assay.

**Results:**

Severe loss of HT29 cell viability was detected for 50 μg/ml EcEE immediately after 24 h exposure whereas the lower concentrations assayed (0.5, 5 and 25 μg/ml) resulted in significant viability decreases after 96 h. Exposure to 25 μg/ml EcEE for 48 h resulted in irreversible cell damage leading to a drastic decrease in cell viability after 72 h recovery in EcEE-free medium. 48 h 25 μg/ml EcEE treatment also induced alteration of colony morphology, H3K9 hyperacetylation, transcriptional up regulation of *p21* and down regulation of *NCL, FOS* and *AURKA*, indicating reduced proliferation capacity. This treatment also resulted in drastic mitotic and nuclear disruption accompanied by up-regulation of *bcl-xl*, limited TUNEL labeling and nuclear size increase, suggestive of a non-apoptocic cell death pathway. EcEE/BPA co-exposure increased mitotic anomalies particularly for the lowest EcEE concentration, although without major effects on viability. Conversely, EcEE/DOX co-exposure decreased cell viability in relation to DOX for all EcEE concentrations, without affecting the DOX-induced cell cycle arrest.

**Conclusions:**

EcEE has cytotoxic activity on HT29 cancer cells leading to mitotic disruption and non-apoptotic cell death without severe induction of DNA damage. Interaction experiments showed that EcEE can increase BPA aneugenic effects and EcEE synergistic effects with DOX supporting a potential use as adjuvant in chemotherapeutic approaches.

## Background

*Eupatorium cannabinum L.*, commonly known as hemp-agrimony is a robust perennial herbaceous plant of the Asteraceae family and the only species of the Eupatorium genus found in Europe occurring also throughout North Africa and Asia [1]. *E. cannabinum* has long been used for medicinal purposes being referred to by Greeks and Romans as well by the medieval Persian physician Aviccena, for what is also known as Eupatorium of Aviccena, and later by the Portuguese Renaissance pioneer in tropical medicine, Garcia da Orta (1563) [2]. Presently, hemp-agrimnony is used in both Chinese [3] and Indian [4] traditional medicine as well as in natural medicine in western countries [5] with very diverse therapeutic indications including influenza-like illnesses [6], hypertension [3, 4, 6] and as an anti-tumour agent [4]. *E. cannabinum* extracts has been previously characterized and reveal the presence of sesquiterpenes [7], pyrrolizidine alkaloids [3, 8] as well as several phenolic compounds [9, 10].

Sesquiterpenes were found to be a major fraction (43.3%) of essential oil from *E. cannabinum* aerial parts [11], being eupatoriopicrin the main component [7]. Eupatoriopicrin has been associated with induction DNA damage in Ehrlich ascites tumour [12] as well as with cytostatic activity and both *in vitro* and *in vivo* tumour growth inhibition properties in Lewis lung carcinoma and FIG 26 fibrosarcoma [13].

Pyrrolizidine alkaloids are generally associated with genotoxicity and tumourigenic activities [14], however the isomers intermedine and lycopsamine indentified in *E. cannabinum* have low genotoxic potency [15] and lycopsamine was shown to be non-tumourigenic in rats [16]. Additionally the phenolic compounds identified in this plant have been described to have anti-inflammatory [9], anti-parasitary [17], as well as anti-proliferative effects in several cell lines [18]. In particular, jaceosidin cytotoxic effects have been demonstrated in normal and cancer endometrial cells [19] and hispidulin was shown to efficiently inhibit growth of gastric cancer cells [20] and liver carcinoma cells without significant toxic effect in normal liver cells [21].

Although the effects of specific components of *Eupatorium cannabinum L.* extracts have been described, the cellular effects of the full extracts have not, until now, been investigated. Thus, here different concentrations of *Eupatorium cannabinum L.* ethanolic extract (EcEE) were evaluated on the colon cancer cell line HT29. Moreover we also analyzed its interactions with the synthetic phenolic compound bisphenol A (BPA) as well as with the chemotherapeutic agent Doxorubicin (DOX). Human exposure to BPA is considered generalized in the common population and its adverse health effects are the focus of intense investigation [22, 23]. On the other hand, DOX is a commonly used chemotherapeutic agent to which cell resistance can emerge [24, 25]. Plant constituents are a major source of bioactive compounds and several plants have been investigated aiming to identify potential synergistic effects with DOX (reviewed in [26]).

## Methods

### *Eupatorium cannabinum L.*ethanolic extract

*Eupatorium cannabinum* L. (Asteraceae) aerial parts were collected in the Rossas fields of Arouca village, Portugal, in August during mass flowering. Formal identification of plant material was performed by A.P. Paes from “João de Carvalho e Vasconcellos Herbarium” at Instituto Superior de Agronomia (Lisboa, Portugal). A voucher specimen was deposited in the same herbarium under the number LISI 1503/2013. Plant material was dried and powdered using a grinder and ethanolic extract (EcEE) was obtained by soaking the material in absolute ethanol for 48 h at room temperature with gentle shaking. The extract were filtered and concentrated under vacuum on a rotary evaporator at 40°C and stored at -20°C for further use.

### Cellular cultures, reagents and treatments

HT29 cells were purchased from European Collection of Cell Cultures (ECACC, UK) and cultivated in RPMI medium under standard conditions as previously described [27]. Before treatments and experiments HT29, cells were allowed to stabilize for 24 h in standard medium and further cultivated in EcEE supplemented media for 24 h, 48 h or 96 h. Crude ethanolic extract was dissolved in ethanol to a final work concentration of 50 mg/ml before use and added to the culture media at four different final concentrations (0.5 μg/ml, 5 μg/ml, 25 μg/ml and 50 μg/ml). Bisphenol A (Sigma-Aldrich) was freshly diluted in ethanol and added to the culture media to the final concentration of 1 μg/ml (4.4 μM) that corresponds to the established Tolerable Daily Intake (TDI) level of 50 ug/kg BW/day [28, 29] considering an average body weight of 70 Kg and daily consumption of 3 litres of preformed water. Doxorubicin (DOX) (AppliChem) was dissolved in water at stock concentration of 1 mg/ml and added to the culture media to final concentration of 2.5 μg/ml (4 μM) which corresponds to a therapeutic dosage [30]. For the combined EcEE/BPA or EcEE/DOX exposures, cells were pre-exposed to EcEE for 24 h followed by additional 24 h of simultaneous exposure to EcEE and BPA or EcEE and DOX. Single 24 h BPA or DOX exposure was carried-out in equivalent cell cultures. For evaluation of cell recovery capacity after treatments cells were cultivated for additional 72 h in standard culture medium. Negative controls were performed for all experiments using cells grown in standard culture medium as well as cells grown in medium supplemented with ethanol at final concentration of 170 μM, corresponding to the final concentration of ethanol used as vehicle for all EcEE concentrations as well as for BPA.

### Cell viability

Cell viability was evaluated by CellTiter-Blue assay (Promega) following manufacturer’s instructions. Cells were plated on 96-well plates at a density of 3.2 × 10^4^ cells/well and after treatments were incubated for 4 h with CellTiter-Blue Reagent. Additional negative controls were performed in the absence of cells to guarantee that the utilized media did not interfere with fluorescence readings. Experiments were repeated at least three times with a minimum of three replicates per experiment.

### DAPI staining, TUNEL assay and immunodetection

For cytological analysis cells were grown over glass coverslips coated with 0.2% (v/v) gelatin (Sigma-Aldrich) and after treatments fixed in 4% (p/v) formaldehyde in PBS. For evaluation of colony morphology, mitotic index as well as mitotic and nuclear anomalies cells were DAPI stained and mounted on glass slides with antifade AF1 (Citifluor). DNA damage assessment with TUNEL assay (Roche) was performed accordingly to manufacturers’ instructions. Immunodetection of H3K9ac and α-tubulin was performed in fixed cells as previously described [27] using the primary antidodies anti-acetyl-histone H3(Lys 9) (ab10812, Abcam) and anti-α-Tubulin (T9026, Sigma-Aldrich) detected with FITC or Cy3 conjugated secondary antibodies. Images were captured using the appropriate excitation and emission filters and recorded using an epifluorescence microscope Zeiss Axioskop2 equipped with a Zeiss AxioCam MRc5 digital camera. ImageJ software (http://rsbweb.nih.gov/ij/) was used for nuclear area measurements. The analysis was performed in the pooled results of at least two independent experiments with at least two replicates.

### cDNA isolation and real-time quantitative PCR

Transcriptional analysis was performed by quantitative real-time PCR (qRT-PCR) for the proliferation-associated genes nucleolin (*NCL*), *FOS* and *p21*, for the cell cycle related gene *AURKA*, and the anti-apoptotic gene *bcl-xl*. The specific primers utilized are listed in Table 1, *GAPDH* and *β-actin* were used as control genes [27, 31]. Total RNA was extracted from trypsinized cells with the RNAqueous Kit (Invitrogen) following manufacturers’ instructions. 3 μg of total RNA was utilized for RNase free DNase digestion (RQ1 RNase free DNase, Promega) and first strand cDNA synthesis was completed with random primers (DYNAmo cDNA syntesis Kit, Thermo Scientific). The resulting cDNA was utilized for qRT-PCR with SsoFast Eva Green Supermix (BioRad) utilizing the following conditions: 95°C for 3 min, 35 cycles (95°C for 30 s, 55°C for 30 s, 72°C for 40 s), and 72°C for 5 min. Control PCRs were also performed with total RNA prior to cDNA synthesis as well as for all primer combinations without template. Experiments were repeated at least three times with at least three replicates per cell treatment/primer combination in each experiment. Since no significant differences were detected between the two reference genes, threshold cycles (Ct) of the target genes were equilibrated with the mean Ct of GAPDH and β-actin genes to calculate ∆Ct (∆Ct = Ct _target_ – mean Ct _GAPDH:β-actin_). Gene expression levels were analyzed by calculating ∆∆Ct (∆∆Ct = ∆Ct _treatment_ – mean ∆Ct _control_). Results are presented as log2 of the mean fold change (2^-∆∆Ct^) ± standard deviation.

**Table 1:** **Primers used for qRT-PCR**

Sequence	Accession no.	Forward primer (5’ → 3’)	Reverse primer (5´ → 3´)
***p21*** [31]	NM_000380	CTGGAGACTCTCAGGGTCGAA	CCAGGACTGCAGGCTTCCT
***AURKA*** [27]	NM_003600	GCTGGAGAGCTTAAAATTGCAG	TTTTGTAGGTCTCTTGGTATGTG
***FOS*** [31]	NM_005252	AGGAGAATCCGAAGGGAAAG	CAAGGGAAGCCACAGACATC
***bcl-xl*** [31]	NM_001191.2	TTACCTGAATGACCACCTA	ATTTCCGACTGAAGAGTGA
***NCL*** [31]	NM_005381	CCTTCTGAGGACATTCCAAGACA	ACGGTATTGCCCTTGAAATGTT
***GAPDH*** [27]	NM_002046	GAGTCAACGGATTTGGTCGTA	GCAGAGATGATGACCCTTTTG
***β-actin*** [31]	NM_001101	GGTCATCTTCTCGCGGTTGGCCTTGGGGT	CCCCAGGCACCAGGGCGTGAT

### Statistical analysis

Student’s t test was used for statistical analysis of gene transcription, cell viability, nuclear area and nuclear fragmentation. No significant differences were detected between control and vehicle for all parameters analysed, and results are shown in relation to control. GraphPad Prism 6 software was used for determination of IC_50_ values.

## Results

### *E. cannabinum*ethanolic extract decreases HT29 cell viability

**Figure 1:**
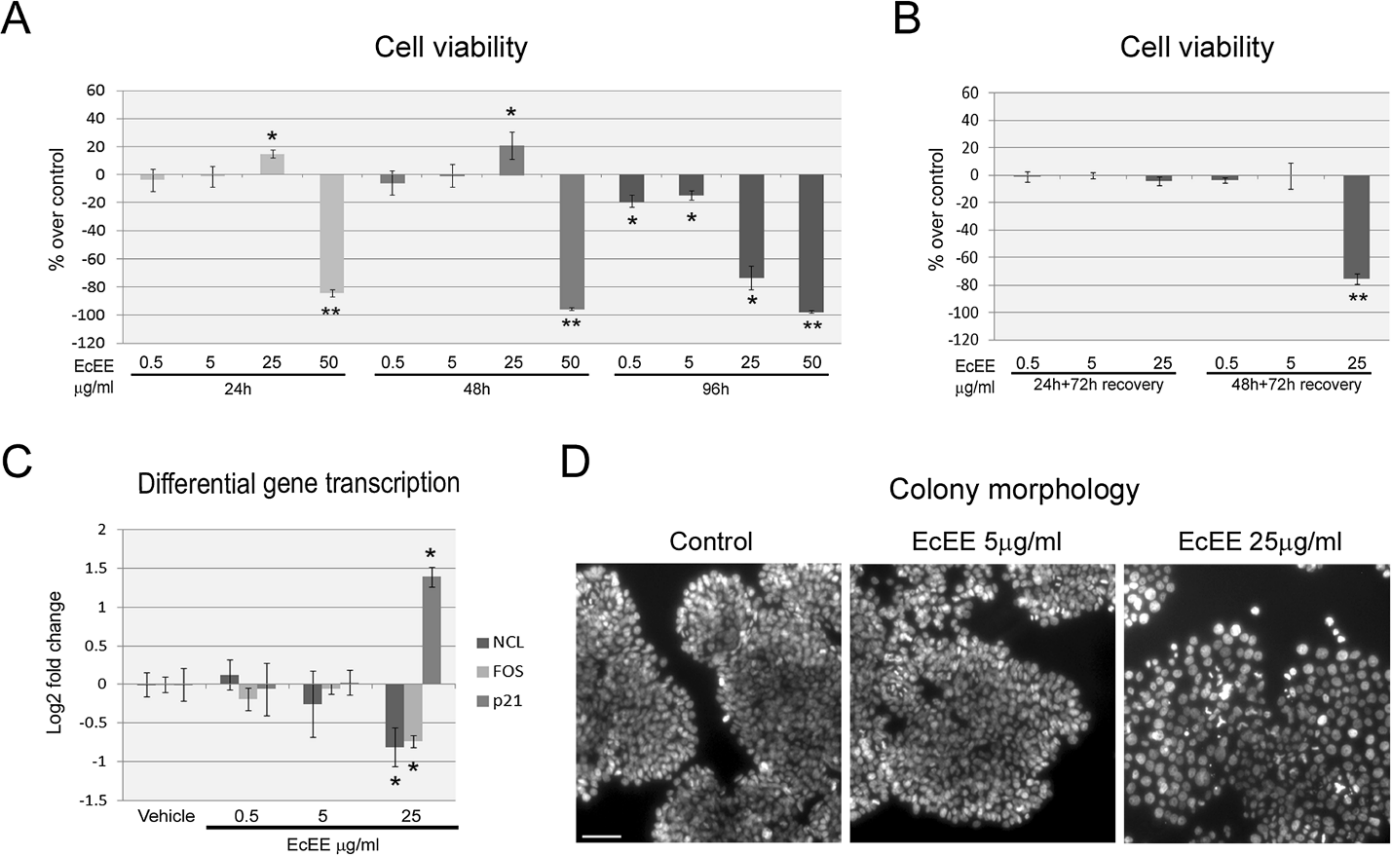
**EcEE affect cell viability and proliferation. (A)** Cell viability after 24 h, 48 h and 96 h of exposure to distinct concentrations of EcEE and **(B)** after 72 h recovery in EcEE-free medium following 24 h and 48 h treatments. Results are presented as percentage over control, **p < 0.0001 and *p < 0.01. **(C)** *NCL*, *FOS* and *p21* differential transcription after 48 h exposure to distinct EcEE concentrations. Results are shown as mean log2 fold change (2^-ΔΔCt^) ± standard deviation in relation control, *p < 0.0001. **(D)** DAPI stained HT29 colonies after 48 h in control medium and medium supplemented with EcEE 5 μg/ml or EcEE 25 μg/ml. All images have identical magnification, bar = 50 μm.

Assessment of cell viability was performed to test potential cytotoxic effects of *E. cannabinum* ethanolic extract (EcEE) on HT29 cells. For this, CellTiter-Blue assay was utilized and effects of different concentrations of EcEE (0.5 μg/ml, 5 μg/ml, 25 μg/ml and 50 μg/ml) were evaluated after 24 h, 48 h and 96 h of exposure (Figure 1-A). The higher EcEE concentration (50 μg/ml) resulted in a severe decrease of cell viability after 24 h exposure and complete loss of viability at subsequent time points analyzed (48 h and 96 h). On the other hand, no decrease in cell viability was detected after 24 h or 48 h for the lower EcEE concentrations, and a slight increase in fluorescence was observed after 24 h and 48 h for 25 μg/ml EcEE. However, significant decreases in cell viability were detected for the three lower EcEE concentrations after 96 h of exposure, and particularly for 25 μg/ml EcEE (-18.89%, -14.55% and -73.25% for 0.5 μg/ml, 5 μg/ml and 25 μg/ml, respectively). Taken together, these results indicate more severe effects after prolonged exposure. This is further shown by IC_50_ values of 46.75, 44.64 and 13.38 μg/ml for 24, 48 and 96 h, respectively.

To detect possible deferred effects of the EcEE exposure cell viability was also evaluated after 72 h of recovery in standard culture media (Figure 1-B) and revealed a severe decrease exclusively for 48 h exposure to 25 μg/ml EcEE (-75.59%).

To better understand the effects of EcEE immediately after 48 h exposure, gene transcription analysis was carried out for three proliferation-associated genes, namely nucleolin (*NCL)*, *p21* and *FOS* (Figure 1-C). Similarly to the cell viability results, no significant differences in transcription levels were detected after 48 h exposure to EcEE concentrations equal to or lower than 5 μg/ml. Conversely, 25 μg/ml EcEE exposure resulted in significant differences in mRNA levels of all three genes, corresponding to down regulation of both *NCL* and *FOS* (Log2 fold change = -0.813 ± 0.248 and -0.741 ± 0.078, respectively), and up regulation of *p21* (Log2 fold change = 1.393 ± 0.128). Evaluation of colony morphology was performed immediately after EcEE treatments by DAPI staining. Again, significant alterations in colony morphology were detected after exposure to 25 μg/ml EcEE for 48 h, evident as cells being more dispersed and showing a flattening of cellular aggregates in comparison to controls with no detectable effect for 5 μg/ml EcEE (Figure 1-D) or 0.5 μg/ml EcEE (not shown).

### *E. cannabinum*ethanolic extract induces alterations in nuclear structure and mitotic disruption

**Figure 2:**
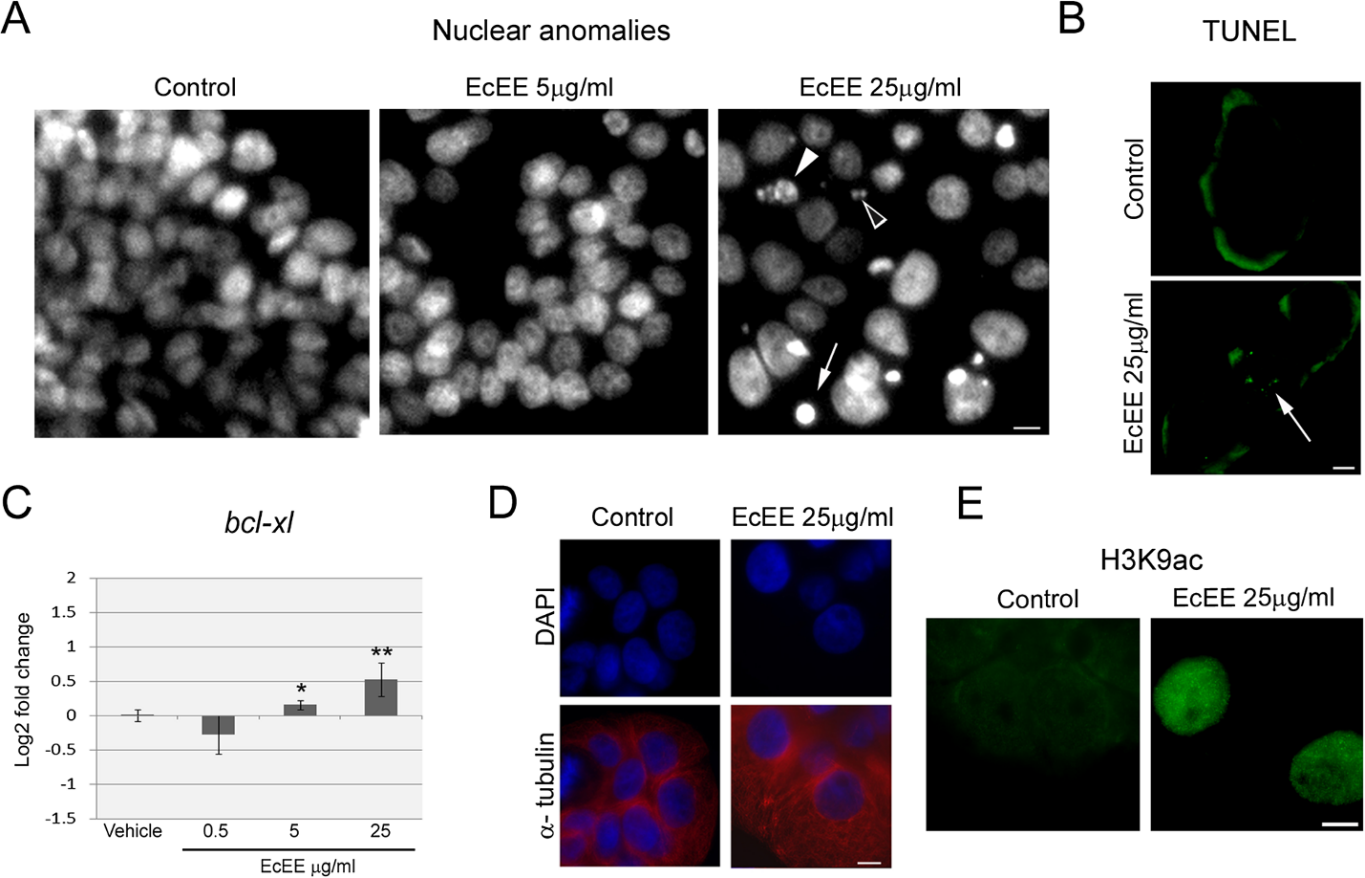
**Nuclear organization is disrupted after 48 h of exposure to EcEE 25 μg/ml. (A)** DAPI stained HT29 interphase cells. Nuclear anomalies, namely pyknosis (arrow), micronuclei (open arrow head) and karyorrhexis (arrow head) are detectable only for 25 μg/ml EcEE. The lack of effects induced by EcEE lower concentrations is exemplified by 5 μg/ml EcEE. **(B)** TUNEL positive nuclei (arrow) with sparse labeling are detectable for 25 μg/ml EcEE. **(C)** *bcl-xl* differential expression after 48 h exposure to distinct EcEE concentrations. Results in are shown as the mean log2 fold change (2^-ΔΔCt^) ± standard deviation in relation control, **p < 0.0001 and *p < 0.01. **(D)** DAPI staining (blue) and α-tubulin immunodetection (red in merged images at the bottom) of interphase cells after 48 h in control or 25 μg/ml EcEE. **(E)** Immunodetection of H3K9ac after 48 h in control or EcEE 25 μg/ml supplemented medium. Within each experiment all images have identical magnification, bar = 5 μm.

A detailed cytological analysis was performed for 0.5 μg/ml, 5 μg/ml and 25 μg/ml EcEE concentrations after 48 h of exposure and again significant nuclear alterations were observed exclusively for 25 μg/ml EcEE (Figure 2-A). This was obvious as the prominent occurrence of micronuclei and highly condensed nuclei (pyknosis) scattered throughout cell aggregates as well as fragmented nuclei (karyorrhexis), revealing irreversible nuclear damage. In addition, TUNEL assay showed that induction of DNA breaks also occurred after 48 h exposure to 25 μg/ml EcEE treatments although at a much lower level than nuclear abnormalities, as many of the abnormal nuclei were not TUNEL positive and in positive nuclei labeling was sparse (Figure 2-B). For the two lower EcEE concentrations (0.5 and 5 μg/ml) no TUNEL positive nuclei were detected (not shown) as observed for control. Importantly, qRT-PCR transcriptional analysis of the anti-apoptotic *bcl-xl* gene revealed that EcEE exposure induced a significant up regulation of this gene not only at 25 μg/ml (Log2 fold change = 0.528 ± 0.243) but also at 5 μg/ml, although to a lesser extent (Log2 fold change = 0.158 ± 0.067) (Figure 2-C). Quantification of the nuclear area of non-pyknotic and non-fragmented DAPI stained nuclei showed a significant increase in this parameter in relation to control for cells exposed to 25 μg/ml EcEE but not to the lower EcEE concentrations (not shown). The increment in nuclear area after the 48 h exposure to 25 μg/ml EcEE corresponded in average to 48.8% (n > 70 for each growth condition, p < 0.0001 for 25 μg/ml EcEE in relation to control) and was accompanied by an evident increase in cellular area revealed by α-tubulin immunodetection (Figure 2-D). Moreover, evident chromatin enrichment in histone H3 acetylated on lysine 9 (H3K9ac) was detected also for 48 h 25 μg/ml EcEE (Figure 2-E) whereas no alteration was observed for either 0.5 μg/ml or 5 μg/ml EcEE (not shown).

**Figure 3:**
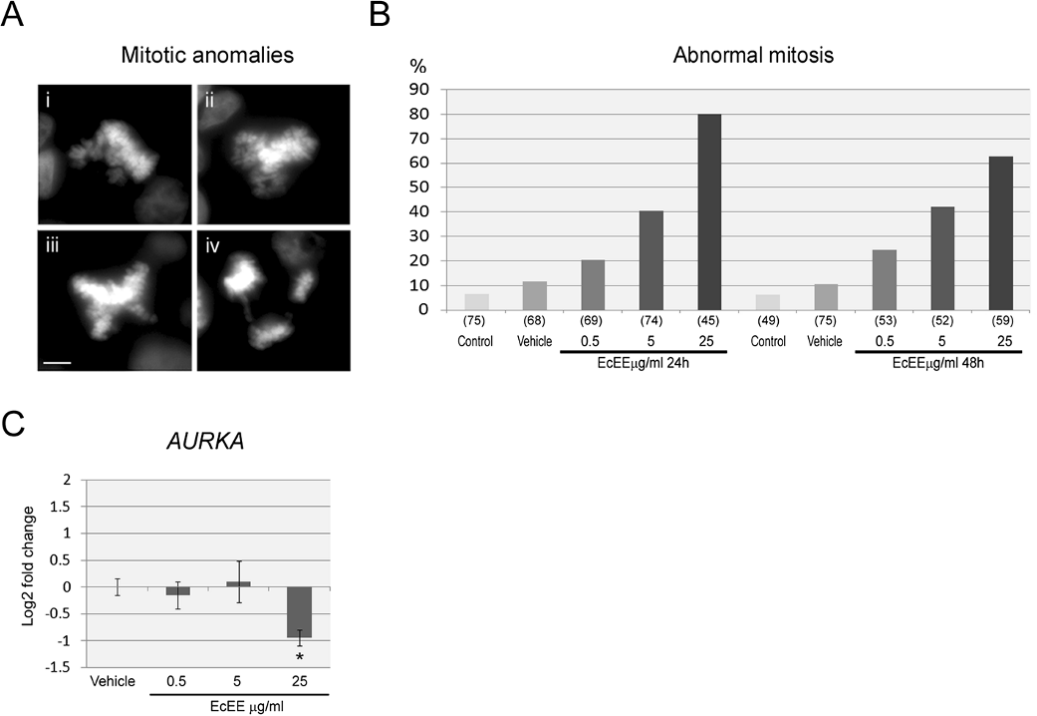
**EcEE exposure induces mitotic disruption. (A)** DAPI stained abnormal mitotic cells after EcEE exposure showing (i) defective chromosome congression, (ii) tripolar and (iii) tetrapolar metaphases and (iv) tripolar anaphase with chromosome bridges, bar = 5 μm. **(B)** Percentage of abnormal mitosis after 24 h and 48 h exposure to distinct concentrations of EcEE; the total number of mitotic cells scored and utilized to calculate the percentage of abnormal mitosis is shown in brackets. **(C)** *AURKA* differential transcription after exposure to 48 h of EcEE. Results are shown as the mean log2 fold change (2^-ΔΔCt^) ± standard deviation in relation control, *p < 0.0001.

The effects of exposure to EcEE were further evaluated on mitotic cells after DAPI staining. No significant variation was observed in the mitotic index between control, vehicle and EcEE independently of the concentration assayed (varying between 4.57 and 5.94). On the other hand, although mitotic anomalies, particularly multipolar metaphases and anaphases, are a common feature of HT29 cells and therefore observed both in control and vehicle (6.67% and 11.76% after 24 h; 6.38% and 10.67% after 48 h, for control and vehicle respectively), the percentage of abnormal mitosis increased after exposure to all EcEE concentrations (Figures 3-A and B). Although a slight increase of abnormal mitosis was already detectable for 0.5 μg/ml EcEE, this effect was greater for 5 μg/ml EcEE (41% and 44% after 24 h or 48 h, respectively). After exposure to 25 μg/ml EcEE, most mitotic cells presented abnormalities (80% and 63% after 24 and 48 h, respectively). Although the frequency of abnormal mitosis was greater after 24 h at the higher EcEE concentration, these results clearly indicate that EcEE induces mitotic disruption in a dose dependent manner. Interestingly, qRT-PCR analysis revealed a significant down regulation of *AURKA* (Log2 fold change = -0.938 ± 0.146), a gene that encodes a key protein for mitotic chromosome segregation (Figure 3-C).

### *E. cannabinum*ethanolic extract increases Bisphenol A induced mitotic disruption

**Figure 4:**
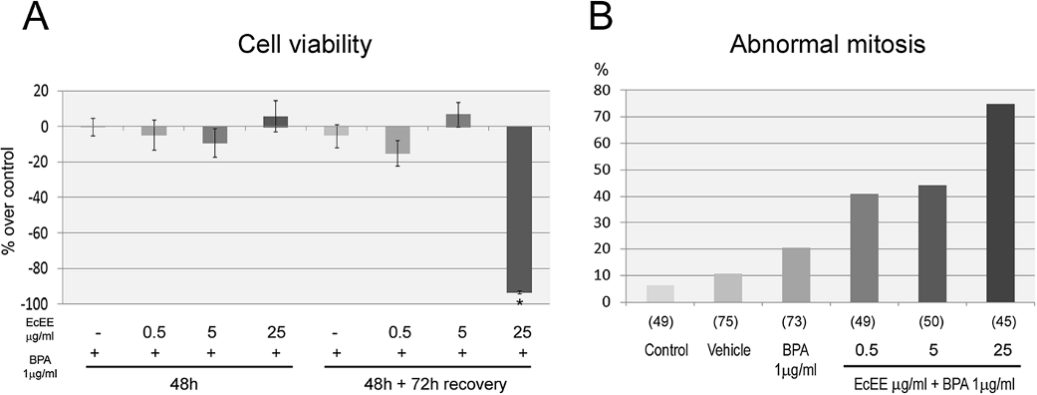
**EcEE interacts with BPA at reference level. (A)** Cell viability after co-exposure to BPA (1 μg/ml) and distinct EcEE concentrations and subsequent 72 h recovery in standard medium. Results are presented as percentage over control, *p < 0.001. **(B)** Percentage of mitotic anomalies after 48 h culture in standard medium (control) or medium supplemented with ethanol (vehicle), BPA or EcEE at distinct concentrations in combination with BPA. The total number of mitotic cells scored and utilized to calculate the percentage of abnormal mitosis is shown in brackets.

Interactions between EcEE and the environmental pollutant BPA at reference level (1 μg/ml) were evaluated. Co-exposure to EcEE and BPA did not affect cell viability immediately after treatments, as no significant differences were detected in relation to control (Figure 4-A). After 72 h recovery in standard medium, a severe decrease in cell viability (-93.48%) was exclusively observed for 25 μg/ml EcEE/BPA (Figure 4-A) which was even greater than that observed for 25 μg/ml EcEE alone (Figure 1-B).

Cytological evaluation of mitotic disruption after DNA DAPI staining (Figure 4-B) revealed that BPA exposure alone increased the level of mitotic anomalies to 20.5%. Interestingly, a stronger effect of BPA co-exposure was observed for the lowest EcEE concentration assayed (0.5 μg/ml), which resulted in 41% of abnormal mitosis (Figure 4-B) compared to 25% observed for EcEE alone (Figure 3-B). In contrast, no evident effect of BPA was detected for the intermediate EcEE concentration, evident as an identical level of 44% for 5 μg/ml EcEE alone or in combination with BPA. Co–exposure to the higher EcEE concentration (25 μg/ml) and BPA resulted in a particular high level of mitotic anomalies (75%), although the difference in relation to EcEE alone (63%) was smaller than that observed for the lower EcEE concentrations.

### Cytotoxic effects of Doxorubicin are enhanced by *E. cannabinum*

**Figure 5:**
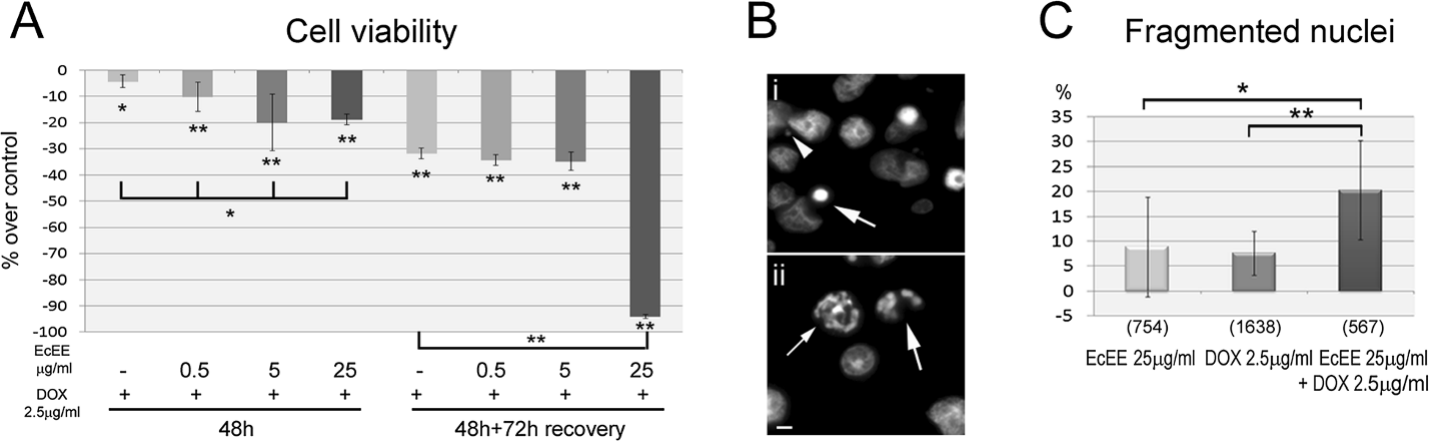
**EcEE has synergistic effects with DOX at a therapeutic dose. (A)** Cell viability after co-exposure to DOX (2.5 μg/ml) and distinct EcEE concentrations and subsequent 72 h recovery in standard medium. Results are presented as percentage over control, the level of significance in relation to DOX alone is indicated by horizontal brackets, **p < 0.0001 and *p < 0.01. **(B)** DAPI stained cell after co-exposure to EcEE 25 μg/ml/DOX illustrating the occurrence of (i) micronuclei (arrow head) and pyknotic nuclei (arrow) and (ii) fragmented nuclei (arrows), bar = 5 μm. **(C)** Percentage fragmented nuclei after exposure to EcEE 25 μg/ml or DOX alone and the combination of both. Total number of cells analyzed is shown in brackets, **p < 0.0001 and *p < 0.03 in relation to EcEE 25 μg/ml or DOX alone.

Potential interactions between different concentrations of EcEE and the chemotherapeutic drug doxorubicin (DOX) at a therapeutic concentration of 2.5 μg/ml were investigated. Immediately after exposure, DOX alone resulted in a slight decrease in cell viability (-4.15%) (Figure 5-A). Interestingly, the loss of cell viability was significantly more pronounced after co-exposure to EcEE/DOX for all EcEE concentrations (-10.03%, -19.88% and -18.67% for 0.5 μg/ml, 5 μg/ml and 25 μg/ml, respectively) (Figure 5-A) contrasting with the lack of effects observed for 48 h exposure to EcEE alone (Figure 1-A). Recovery experiments showed that the effects of both DOX and 25 μg/ml EcEE/DOX have long lasting negative effects on viability, apparent as prominent decreases in cell viability after 72 h recovery in standard medium in relation to what was observed immediately after exposure (Figure 5-A). After recovery, EcEE 25 μg/ml/DOX exposure resulted in an even more pronounced loss of cell viability (-93.95%) than that observed for exposure to 25 μg/ml EcEE alone (Figure 1-B). Conversely, for the lower EcEE concentrations, no significant differences were detected between exposure to DOX alone and in combination with EcEE (Figure 1-B).

Cytological analysis after DAPI staining showed a complete absence of cells at mitosis after exposure to DOX alone or in combination to EcEE, independently of the EcEE concentration. Conversely, both pyknotic cells and fragmented nuclei were observed after exposure to DOX alone or in combination to EcEE (Figure 5-B). Since identical nuclear alterations were also observed after single exposure to 25 μg/ml EcEE (Figure 2-E), the levels of nuclear fragmentation were compared between single exposure to 25 μg/ml EcEE or DOX alone and the combination of both (Figure 5-C). The results revealed that the induction of nuclear fragmentation is significantly higher for 25 μg/ml EcEE/DOX combined exposure (20.28%) than for either DOX (7.63%) or 25 μg/ml EcEE (8.89%) alone.

## Discussion

*Eupatorium cannabinum* L. is a commonly utilized plant for alternative and/or complementary medicine treatments [6] including as an anticancer agent [4]. Although cellular effects of particular phytochemicals known to be present in *E. cannabinum* have been previously described, to our knowledge this is the first study that evaluates the cytotoxic potential of *E. cannabinum* extracts on human cancer cells. Here we demonstrated that *E. cannabinum* ethanolic extract (EcEE) has cytotoxic effects on HT29 colon cancer cells in a time and dose dependent manner. IC_50_ were similar after 24 and 48 h (46.75 and 44.65 μg/ml, respectively) but considerably lower (13.38 μg/ml) after 96 h of exposure. Cytotoxic activity has also been demonstrated for extracts from other *Eupatorium* species. For *E. perfoliatum* ethanolic extract, IC_50_ values between 12 and 14 μg/ml were obtained after 24 h exposure in three distinct mammalian cell lines [32]. In MCF7 breast cancer cells a time dependent effect was also observed for *E. odoratum* ethyl acetate extract (IC_50_ of 65.72, 83.88 μg/ml and 92.84 μg/ml for 24, 48 and 72 h, respectively) while for acetone extract higher IC_50_ values were obtained but without a direct correlation with exposure time (133.9, 163.0 and 147.8 μg/ml for 24, 48, and 72 h respectively) [33]. The immediate cytotoxicity observed here for EcEE is lower than that obtained for *E. perfoliatum* ethanolic extract and higher than that of ethyl acetate or acetone extracts from *E. odoratum*. Interestingly the time dependent increase in cytotoxicity of EcEE was only detected for the longer exposure time (96 h). Moreover, a deferred effect on cell viability was detected after 48 h exposure to EcEE at 25 μg/ml. This was also associated with disruption of cell colony three-dimensional arrangement, a generalized increase in nuclear area and H3K9 hyperacetylation. Relevantly, gene transcription analysis revealed a significant reduction in the mRNA levels of *FOS*, which encodes for a nuclear protein from AP-1 transcription factor complex, and nucleolin (*NCL*) the most profuse non-ribosomal protein of the nucleolus. Both *FOS* and nucleolin are involved in the regulation of cell proliferation [34, 35] as their decreased expression has been related with reduced proliferation capacity of cancer cells including colon cancer cell lines [36, 37]. On the other hand, exposure to EcEE (25 μg/ml, 48 h) also resulted in the up regulation of *p21*, a cyclin-dependent kinase inhibitor which is a major regulator of the cell cycle [38]. It was previously shown that histone hyperacetylation induces *p21* over expression [39]. In colon cancer cells inhibition of histone deacetylation results in both up regulation of *p21* [40], and induction of G2/M cell cycle arrest [41]. Relevantly, cell reduction capacity depends on the cell cycle being higher at G2/M [42]. Considering that the cell viability assay used is based on the resazurin reduction and that overall our results were incompatible with EcEE induction of cell proliferation, the slight and transient augment of fluorescence detected after 24 h and 48 h of exposure to 25 μg/ml EcEE was also suggestive of cell arrest at G2 or M. Moreover, the increase of abnormal mitotic cells after exposure to EcEE is also suggestive of a mitotic block. This phenotype was accompanied by a significant down regulation of Aurora A transcription, which is consistent with previous results showing that decreased Aurora A levels are associated with mitotic catastrophe and consequent cell death [43]. Induction of cell death after 48 h exposure to 25 μg/ml was evident by the prominent occurrence of pyknotic and fragmented nuclei, characteristic of both apoptotic as well as necrotic cells, and supports the marked loss in cell viability observed after recovery. This was moreover associated with transcriptional up regulation of the anti-apoptotic gene *bcl-xL* suggesting a non-apoptotic cell death pathway [44] which is also supported by limited occurrence of DNA breaks. These observations together with the increase in cell size is compatible with a necrotic cell death or necroptosis, a process which acts as backup death-inducing mechanism when apoptosis is inhibited [45].

Cytostatic activity was previously described for compounds identified in *E. cannabinum* extracts, namely the sesquiterpene eupatoriopicrin [7] and the flavonoids centaureidin, jaceosidin and hispidulin [10]. Severe decrease of tumour cell survival *in vitro* was associated with eupatoriopicrin concentrations ranging from 1–10 μg/ml [12, 13] which was correlated with induction of DNA damage [12]. Also, anti-proliferative effects on distinct cancer cell lines have been described for centaureidin concentrations below 1 μg/ml [18] as well as for jaceosidin in the concentration range of 20–50 μg/ml [19] and hispidulin for 4–30 μg/ml [21]. Relevantly, both jaceosidin [19] and hispidulin [20] effects were associated with increased *p21* expression. The results obtained here indicate that the anti-proliferative potency of EcEE is similar to that observed for some of its individual constituents such as eupatoriopicrin, jaceosidin and hispidulin, albeit without marked induction of DNA damage and therefore suggesting a combined action of distinct compounds.

Importantly, EcEE combined exposures with DOX at therapeutic concentration resulted in a clear enhancement of cytotoxic effects, evident as combined treatments significantly decreasing HT29 cell viability immediately after exposure, even for the lower EcEE concentration that *per se* did not affect cell viability. This was accompanied by increased nuclear fragmentation and reduced cell survival after recovery resulting in almost total loss of cell viability. DOX is a commonly utilized antineoplastic drug that acts in tumour cells by induction of apoptosis [46]. Nevertheless different types of cell death can occur simultaneously, independently or through partially common pathways (reviewed in [45]). The severe decrease in cell viability observed after combined exposure to DOX and EcEE can thus result from induction of distinct cell death mechanisms. On the other hand therapeutic concentrations of DOX induces cell arrest at G2/M and/or G1/S checkpoints [47, 48]. The results obtained show that EcEE does not counteract DOX-induced cell cycle arrest. Considering that DOX acts by induction of apoptosis [46] to which cell resistance can emerge [24, 25] our data substantiates potential adjuvant EcEE properties in chemotherapeutic approaches [49].

On the other hand, no immediate effect on cell viability was associated with co-exposure to EcEE and the synthetic phenolic compound BPA. However, cell recovery capacity after 48 h exposure to 25 μg/ml EcEE decreased by the presence of BPA. Additionally, EcEE/BPA combined exposures resulted in increased mitotic anomalies in relation to either BPA or EcEE alone for 25 μg/ml EcEE but also for 0.5 μg/ml EcEE. BPA is characterized as an aneugenic chemical [50] capable of interfering with cell division mechanisms even at very low concentrations [27]. Nonetheless BPA is widely used in a variety of consumer products leading to a generalized human exposure although its risks remain highly controversial [23]. The present results raise the possibility that adverse BPA effects could be enhanced by interactions with other chemicals, an aspect that remains largely unknown and has barely been addressed.

## Conclusions

*E. cannabinum* has been utilized as a medicinal plant for alternative and/or complementary medicine, however the effects or the mode of action of full extracts have not been evaluated at the cellular level. The present work demonstrates that *E. cannabinum* ethanolic extract has potent cytotoxic activity against HT29 colon cancer cells associated with mitotic disruption and cell death without marked evidences of DNA damage. Relevantly *E. cannabinum* extract exhibits synergistic effects with doxorubicin in the induction of HT29 cell death indicating its potential use in alternative or complementary therapeutic strategies. On the other hand, the results show also that *E. cannabinum* can increase aneugenic effects of the environmental pollutant BPA, drawing attention to the possibility that BPA adverse effects may be potentiated by interaction with other chemicals.

## References

[1] Schmidt GJ, Schilling EE (2000). Phylogeny and biogeography of Eupatorium (Asteraceae: Eupatorieae) based on nuclear ITS sequence data. Am J Bot.

[2] da Orta G (1913). Colloquies on the simples & drugs of India; translated with an introduction and index by Sir Clements Markham.

[3] Fu PP, Yang Y, Xia Q, Chou MW, Cui YY, Lin G (2002). Pyrrolizidine Alkaloids - Tumorigenic Components in Chinese Herbal Medicines and Dietary Supplements. J Food Drug Anal.

[4] Roeder E, Wiedenfeld H (2013). Plants containing pyrrolizidine alkaloids used in the traditional Indian medicine-including ayurveda. Pharmazie.

[5] Kozel C (1982). Guía de medicina natural Vol II Plantas medicinales.

[6] Jaric S, Popovic Z, Macukanovic-Jocic M, Djurdjevic L, Mijatovic M, Karadzic B, Mitrovic M, Pavlovic P (2007). An ethnobotanical study on the usage of wild medicinal herbs from Kopaonik Mountain (Central Serbia). J Ethnopharmacol.

[7] Rucker G, Schenkel EP, Manns D, Mayer R, Hausen BM, Heiden K (1997). Allergenic sesquiterpene lactones from Eupatorium cannabinum L. and Kaunia rufescens (Lund ex de Candolle). Nat Toxins.

[8] Boppre M, Colegate SM, Edgar JA, Fischer OW (2008). Hepatotoxic pyrrolizidine alkaloids in pollen and drying-related implications for commercial processing of bee pollen. J Agric Food Chem.

[9] Chen JJ, Tsai YC, Hwang TL, Wang TC (2011). Thymol, benzofuranoid, and phenylpropanoid derivatives: anti-inflammatory constituents from Eupatorium cannabinum. J Nat Prod.

[10] Zhang ML, Wu M, Zhang JJ, Irwin D, Gu YC, Shi QW (2008). Chemical constituents of plants from the genus Eupatorium. Chem Biodivers.

[11] Paolini J, Costa J, Bernardini AF (2005). Analysis of the essential oil from aerial parts of Eupatorium cannabinum subsp. corsicum (L.) by gas chromatography with electron impact and chemical ionization mass spectrometry. J Chromatogr A.

[12] Woerdenbag HJ, van der Linde JC, Kampinga HH, Malingre TM, Konings AW (1989). Induction of DNA damage in Ehrlich ascites tumour cells by exposure to eupatoriopicrin. Biochem Pharmacol.

[13] Woerdenbag HJ, Lemstra W, Malingre TM, Konings AW (1989). Enhanced cytostatic activity of the sesquiterpene lactone eupatoriopicrin by glutathione depletion. Br J Cancer.

[14] Fu PP, Xia Q, Lin G, Chou MW (2004). Pyrrolizidine alkaloids-genotoxicity, metabolism enzymes, metabolic activation, and mechanisms. Drug Metab Rev.

[15] Chen T, Mei N, Fu PP (2010). Genotoxicity of pyrrolizidine alkaloids. J Appl Toxicol.

[16] Xia Q, Zhao Y, Von Tungeln LS, Doerge DR, Lin G, Cai L, Fu PP (2013). Pyrrolizidine alkaloid-derived DNA adducts as a common biological biomarker of pyrrolizidine alkaloid-induced tumorigenicity. Chem Res Toxicol.

[17] Sulsen VP, Cazorla SI, Frank FM, Redko FC, Anesini CA, Coussio JD, Malchiodi EL, Martino VS, Muschietti LV (2007). Trypanocidal and leishmanicidal activities of flavonoids from Argentine medicinal plants. Am J Trop Med Hyg.

[18] Forgo P, Zupko I, Molnar J, Vasas A, Dombi G, Hohmann J (2012). Bioactivity-guided isolation of antiproliferative compounds from Centaurea jacea L. Fitoterapia.

[19] Lee JG, Kim JH, Ahn JH, Lee KT, Baek NI, Choi JH (2013). Jaceosidin, isolated from dietary mugwort (Artemisia princeps), induces G2/M cell cycle arrest by inactivating cdc25C-cdc2 via ATM-Chk1/2 activation. Food Chem Toxicol.

[20] Yu CY, Su KY, Lee PL, Jhan JY, Tsao PH, Chan DC, Chen YL (2013). Potential Therapeutic Role of Hispidulin in Gastric Cancer through Induction of Apoptosis via NAG-1 Signaling. Evid Based Complement Alternat Med.

[21] Gao H, Wang H, Peng J (2014). Hispidulin Induces Apoptosis Through Mitochondrial Dysfunction and Inhibition of P13k/Akt Signalling Pathway in HepG2 Cancer Cells. Cell Biochem Biophys.

[22] Vandenberg LN, Maffini MV, Sonnenschein C, Rubin BS, Soto AM (2009). Bisphenol-A and the Great Divide: A Review of Controversies in the Field of Endocrine Disruption. Endocr Rev.

[23] Vandenberg LN, Chahoud I, Heindel JJ, Padmanabhan V, Paumgartten FJ, Schoenfelder G (2010). Urinary, circulating, and tissue biomonitoring studies indicate widespread exposure to bisphenol A. Environ Health Perspect.

[24] Riganti C, Doublier S, Viarisio D, Miraglia E, Pescarmona G, Ghigo D, Bosia A (2009). Artemisinin induces doxorubicin resistance in human colon cancer cells via calcium-dependent activation of HIF-1alpha and P-glycoprotein overexpression. Br J Pharmacol.

[25] Doublier S, Riganti C, Voena C, Costamagna C, Aldieri E, Pescarmona G, Ghigo D, Bosia A (2008). RhoA silencing reverts the resistance to doxorubicin in human colon cancer cells. Mol Cancer Res.

[26] Kapadia GJ, Rao GS, Ramachandran C, Iida A, Suzuki N, Tokuda H (2013). Synergistic cytotoxicity of red beetroot (Beta vulgaris L.) extract with doxorubicin in human pancreatic, breast and prostate cancer cell lines. J Complement Integr Med.

[27] Ribeiro-Varandas E, Viegas W, Pereira HS, Delgado M (2013). Bisphenol A at concentrations found in human serum induces aneugenic effects in endothelial cells. Mutat Res.

[28] EFSA – European Food Safety Authority (2006). Opinion of the Scientific Panel on Food Additives, Flavourings, Processing Aids and Materials in Contact with Food on a request from the Commition related to 2.2 - BIS(4-HYDROXYPHENYL) PROPANE (bisphenol A). EFSA J.

[29] EFSA – European Food Safety Authority (2010). Scientific opinion on Bisphenol A:evaluation on a study investigating its neurodevelopmental toxicity, review of recent scientific literature on its toxicity and advice on the Danish risk assessment of Bisphenol A. EFSA J.

[30] Greene RF, Collins JM, Jenkins JF, Speyer JL, Myers CE (1983). Plasma pharmacokinetics of adriamycin and adriamycinol: implications for the design of in vitro experiments and treatment protocols. Cancer Res.

[31] Ribeiro-Varandas E, Pereira HS, Monteiro S, Neves E, Brito L, Ferreira RB, Viegas W, Delgado M (2014). Bisphenol A Disrupts Transcription and Decreases Viability in Aging Vascular Endothelial Cells. Int J Mol Sci.

[32] Habtemariam S, Macpherson AM (2000). Cytotoxicity and antibacterial activity of ethanol extract from leaves of a herbal drug, boneset (Eupatorium perfoliatum). Phytother Res.

[33] Harun FB, Syed Sahil Jamalullail SM, Yin KB, Othman Z, Tilwari A, Balaram P (2012). Autophagic cell death is induced by acetone and ethyl acetate extracts from Eupatorium odoratum in vitro: effects on MCF-7 and vero cell lines. ScientificWorldJournal.

[34] Shaulian E, Karin M (2002). AP-1 as a regulator of cell life and death. Nat Cell Biol.

[35] Mongelard F, Bouvet P (2007). Nucleolin: a multiFACeTed protein. Trends Cell Biol.

[36] Pandey MK, Liu G, Cooper TK, Mulder KM (2012). Knockdown of c-Fos suppresses the growth of human colon carcinoma cells in athymic mice. Int J Cancer.

[37] Turck N, Richert S, Gendry P, Stutzmann J, Kedinger M, Leize E, Simon-Assmann P, Van Dorsselaer A, Launay JF (2004). Proteomic analysis of nuclear proteins from proliferative and differentiated human colonic intestinal epithelial cells. Proteomics.

[38] Xiong Y, Hannon GJ, Zhang H, Casso D, Kobayashi R, Beach D (1993). p21 is a universal inhibitor of cyclin kinases. Nature.

[39] Fang JY, Lu YY (2002). Effects of histone acetylation and DNA methylation on p21(WAF1) regulation. World J Gastroenterol.

[40] Druesne N, Pagniez A, Mayeur C, Thomas M, Cherbuy C, Duee PH, Martel P, Chaumontet C (2004). Diallyl disulfide (DADS) increases histone acetylation and p21(waf1/cip1) expression in human colon tumor cell lines. Carcinogenesis.

[41] Robert V, Mouille B, Mayeur C, Michaud M, Blachier F (2001). Effects of the garlic compound diallyl disulfide on the metabolism, adherence and cell cycle of HT-29 colon carcinoma cells: evidence of sensitive and resistant sub-populations. Carcinogenesis.

[42] Conour JE, Graham WV, Gaskins HR (2004). A combined in vitro/bioinformatic investigation of redox regulatory mechanisms governing cell cycle progression. Physiol Genomics.

[43] Kimura M, Yoshioka T, Saio M, Banno Y, Nagaoka H, Okano Y (2013). Mitotic catastrophe and cell death induced by depletion of centrosomal proteins. Cell Death Dis.

[44] Michels J, Kepp O, Senovilla L, Lissa D, Castedo M, Kroemer G, Galluzzi L (2013). Functions of BCL-X L at the Interface between Cell Death and Metabolism. Int J Cell Biol.

[45] Cerella C, Teiten MH, Radogna F, Dicato M, Diederich M (2014). From nature to bedside: Pro-survival and cell death mechanisms as therapeutic targets in cancer treatment. Biotechnol Adv.

[46] Gamen S, Anel A, Perez-Galan P, Lasierra P, Johnson D, Pineiro A, Naval J (2000). Doxorubicin treatment activates a Z-VAD-sensitive caspase, which causes deltapsim loss, caspase-9 activity, and apoptosis in Jurkat cells. Exp Cell Res.

[47] Bar-On O, Shapira M, Hershko DD (2007). Differential effects of doxorubicin treatment on cell cycle arrest and Skp2 expression in breast cancer cells. Anticancer Drugs.

[48] Lupertz R, Watjen W, Kahl R, Chovolou Y (2010). Dose- and time-dependent effects of doxorubicin on cytotoxicity, cell cycle and apoptotic cell death in human colon cancer cells. Toxicology.

[49] Koehler BC, Jager D, Schulze-Bergkamen H (2014). Targeting cell death signaling in colorectal cancer: current strategies and future perspectives. World J Gastroenterol.

[50] Johnson GE, Parry EM (2008). Mechanistic investigations of low dose exposures to the genotoxic compounds bisphenol-A and rotenone. Mutat Res.

[CR51] The pre-publication history for this paper can be accessed here: http://www.biomedcentral.com/1472-6882/14/264/prepub

